# A new genus and species of bristle worm from Beibu Gulf, South China Sea (Annelida, Polychaeta, Amphinomidae)

**DOI:** 10.3897/zookeys.708.12967

**Published:** 2017-10-16

**Authors:** Yue Sun, Xinzheng Li

**Affiliations:** 1 Institute of Oceanology, Chinese Academy of Sciences, Qingdao 266071, China; 2 University of Chinese Academy of Sciences, Beijing 100049, China; 3 Laboratory for Marine Biology and Biotechnology, Qingdao National Laboratory for Marine Science and Technology, Qingdao, Shandong, 266070, China

**Keywords:** Polychaeta, Amphinomidae, *Alleurythoe*, new genus, new species, South China Sea

## Abstract

*Alleurythoe*, a new genus with type species *Alleurythoe tenuichaeta* **sp. n.**, is described and illustrated based on material from Beibu Gulf, northwestern South China Sea. The new genus is distinguished from all genera within Amphinomidae by a combination of characters: caruncle trilobed, conspicuous, attached to and confluent with the posterior prostomial lobe, which is free from the body wall and has 6-7 folds on each of the lateral lobes; both noto- and neuropodial aciculae are spinose, extending beyond the chaetal lobe. *Alleurythoe tenuichaeta* **sp. n.** is characterized by having branchiae present from chaetiger 4 and a bifurcate neurochaetae capillary. A key distinguishing the genera of Amphinominae is provided.

## Introduction

The Amphinomidae, commonly known as fireworms, are typically associated with rocky and soft bottoms in shallow tropical and subtropical waters (Fauchald 1977; Kudenov 1995). It has been reported that amphinomid chaetae are hollow and filled with complanine, a trimethylamine compound which is transmitted to predators and causes intense irritation through highly brittle, calcareous harpoon notochaetae (Arias 2013; Day 1967; Kudenov 1993; Penner 1970). By contrast, Tilic et al. (2017) showed that chaetae of *Eurythoe complanata* (Pallas, 1766) are not hollow; the skin reactions are upon direct contact injury rather than from venom injections. Amphinomid species have either elongate or fusiform bodies, with caruncles generally extending posteriorly over several anterior chaetigers, branchiae ranging from bipinnate to tufts comprised of digitiform rami, with one dorsal and ventral cirrus per parapodium (Fauchald 1977; Gathof 1984; Kudenov 1995).

According to recent phylogenetic studies (Borda et al. 2012, 2015) Amphinomidae can be subdivided into two subfamilies based on the presence (Archinominae Kudenov, 1991) or absence (Amphinominae Lamarck, 1818) of accessory dorsal cirrus. The subfamily Amphinominae currently includes *Amphinome* Bruguière, 1789, *Cryptonome* Borda, Kudenov, Bienhold & Rouse, 2012, *Eurythoe* Kinberg, 1857, *Hermodice*, Kinberg, 1857, *Hipponoe* Audouin & Milne Edwards, 1830, *Paramphinome* Sars, 1869, and *Pareurythoe* Gustafson, 1930; *Benthoscolex* Horst, 1912, *Branchamphinome* Hartman, 1967, *Linopherus* Quatrefages, 1866, and *Pherecardia* Horst, 1886 are provisionally included.

The purpose of this paper is to describe a new genus and species of Amphinominae based on specimens deposited in the Marine Biological Museum of the Chinese Academy of Sciences. A key distinguishing the genera of the Amphinominae, modified from Borda (2012), is provided.

## Materials and methods

Specimens examined in present paper are deposited in the Marine Biological Museum of the Chinese Academy of Sciences (**MBMCAS**) in the Institute of Oceanology (**IOCAS**), preserved in 75% ethanol solution. A Zeiss Stemi 2000-C stereomicroscope with an AxioCam MRc 5 digital camera was used for observations and drawing.

## Systematics

### Family Amphinomidae Lamarck, 1818

#### Subfamily Amphinominae Lamarck, 1818

##### Genus Alleurythoe gen. n.

Taxon classificationAnimaliaAmphinomidaAmphinomidae

http://zoobank.org/E7B84024-A184-4D44-91AD-DE018B9B70D2

###### Type species.

*Alleurythoe tenuichaeta* sp. n.

###### Diagnosis.

Body elongate, quadrangular. Caruncle trilobed, attached to and confluent with posterior prostomial lobe, free from body wall, median lobe broadly sinusoidal, each lateral lobe with 6-7 folds, supported by a basal plate. Branchiae present from chaetiger 4, dendritically branched. Bifurcate neurochaetae capillary. Both noto- and neuropodial aciculae spinose.

###### Etymology.

The generic name is a combination of the prefix *allo*- (meaning “other” or “alternative” in Greek) and the generic name *Eurythoe*. The new genus is assigned to the subfamily Amphinominae and most similar to *Eurythoe* in morphology. Gender: feminine.

###### Remarks.

*Alleurythoe* gen. n. is assigned to the subfamily Amphinominae Lamarck, 1818 because of the absence of accessory dorsal cirri, and justified as a new genus by the nature of its caruncle (Yáñez-Rivera 2011). The new genus is anatomically similar to *Notopygos* Grube, 1855 and *Chloeia* Lamarck, 1818 in the shape of caruncle which is trilobed and essentially supported by a basal plate. However, in contrast to *Alleurythoe* gen. n., the caruncle in the latter two genera has an elevated median keel with several bilateral folds, and it is usually fused to the body wall on chaetigers 1-2 and free thereafter. In the new genus, median keel of caruncle is broadly sinusoidal, thickened, lacks bilateral folds, and is attached to, and confluent with the posterior prostomial lobe, and free from the body wall. In addition, *Alleurythoe* differs from most other amphinomids in having spinous rather than hastate aciculae, bifurcate neurochaetae capillary, while other amphinomids with heftier bifurcate neurochaetae.

*Alleurythoe* gen. n. is superficially similar to *Eurythoe* Kinberg, 1857 in the shape of caruncle, which in both genera consists of a flattened, pronounced median keel and folded lateral lobes; however, the caruncle of *Eurythoe* Kinberg, 1857 is fused to the body wall for most of its length, the median keel overlaps the lateral lobes, which are scalloped on each side and lack a basal plate (Bindra 1927; Borda 2012; Day 1967). Further, in the new genus, the neurochaetae are capillary (non-spurred or spurred), while the short, thick bifurcate neurochaetae, typical of *Eurythoe* Kinberg, 1857, are absent. An identification key to the genera of Amphinominae modified from Borda (2012) is provided below.

##### Alleurythoe tenuichaeta sp. n.

Taxon classificationAnimaliaAmphinomidaAmphinomidae

http://zoobank.org/6BD0D01F-5705-433A-8ABE-250DA5B6D64B

Figs 1 , 2

###### Material examined.

Holotype, MBM200146, Beibu Gulf, 20°15'N, 109°15'E, 38 meters, mud, coll. Ruiping Sun, 27 August 1962. Paratype: MBM010006, Beibu Gulf, 19°30'N, 108°30'E, 66 meters, mud, coll. Zhengang Fan, 14 May 1960.

###### Measurements.

Holotype incomplete, with anterior fragment and posterior fragment, without posterior end. Anterior fragment with 62 chaetigers, 71 mm long, and 10 mm maximum width, posterior fragment with 50 chaetigers, 60 mm long. Paratype complete, broken into two fragments. Anterior fragment with 60 chaetigers, 75 mm long, and 8 mm maximum width, posterior fragment with 61 chaetigers, 57 mm long.

###### Diagnosis.

Body elongate, quadrangular. Caruncle trilobed, conspicuous, attached to and confluent with posterior prostomial lobe, free from body wall, median lobe broadly sinusoidal, each lateral lobe with 6-7 folds, supported by a basal plate. Parapodia biramous, with thickened collars encompassing noto- and neuropodial fascicular lobes; chaetiger 2 first complete anteriormost annular ring. Branchiae present from chaetiger 4, continuing almost to end of body, dendritically branched. Notochaetae coarser and shorter than neurochaetae, include harpoon chaetae and capillaries; barbs of harpoon chaetae on anteriormost chaetigers absent to few in number, better developed in following chaetigers. Bifurcate neurochaetae capillary.

###### Description.

**Figure 1.**
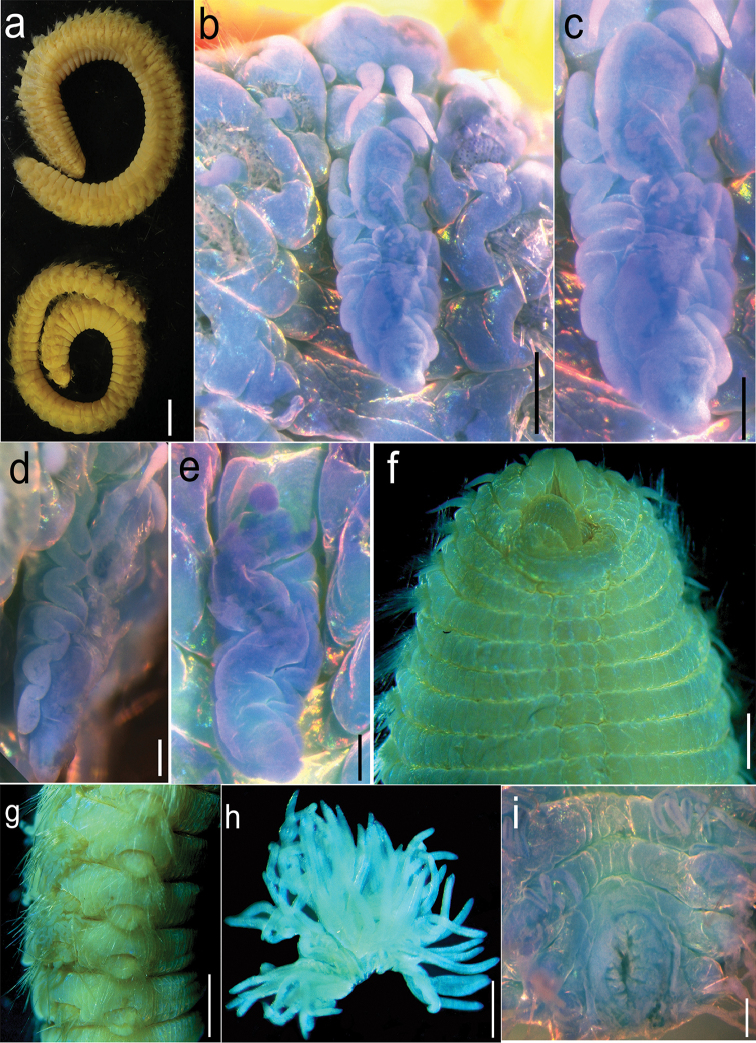
*Alleurythoe tenuichaeta* gen. n. & sp. n., **a** Entire animal in lateral view **b** Prostomium and caruncle, dorsal view **c** Caruncle, dorsal view **d** Caruncle, lateral view. Caruncle, dorsal view **f** Anterior chaetigers, ventral view **g** Parapodia of middle chaetigers, lateral view **h** Branchia of posterior chaetiger **i** pygidium, dorsal view. **a–d, f–i** holotype **e** paratype. Scale bars **a** 0.5 cm; **b** 0.5mm, **c** 0.25 mm; **d–e, i** 0.2 mm; **f** 1 mm; **g** 2 mm; **h** 0.5 mm.

Type specimens preserved alcohol pale, without pigmentation. Body quadrangular in cross section, middle region enlarged, tapering posteriorly (Fig. 1a).

**Figure 2.**
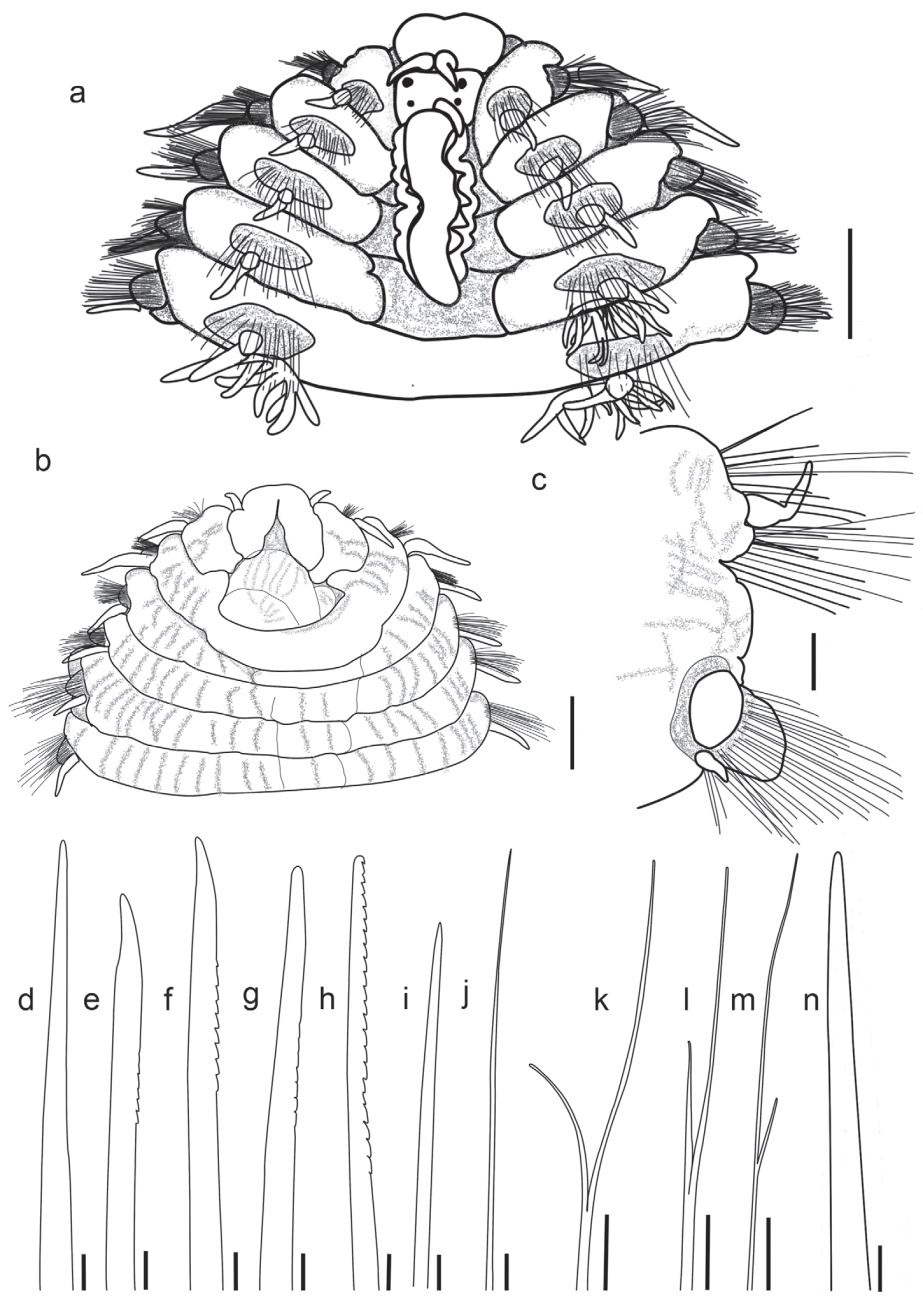
*Alleurythoe tenuichaeta* gen. n. & sp. n., holotype. **a** Anterior chaetigers, dorsal view **b** Anterior chaetigers, ventral view **c** Parapodia of middle chaetiger, posterior view **d** Greatly reduced harpoon chaeta, notopodium of chaetiger 5 **e–g** Reduced harpoon chaeta, notopodium of chaetiger 14 **h** Harpoon chaeta, notopodium of chaetiger 106 **i** Acicula, notopodium of chaetiger 106 **j** Capillary chaeta, neuropodium of chaetiger 85 **k** Bifurcate chaeta, neuropodium of chaetiger 22 **l–m** Bifurcate chaetae, neuropodium of chaetiger 85 **n** Simple chaeta, neuropodium of chaetiger 85. Scale bars: **a–b** 1 mm; **c** 0.5 mm; **d–n** 50 μm.

Prostomium rectangular, divided into two parts by transverse groove. Anterior lobe prominent, slightly bilobed anteriorly, with two palps and two lateral antennae, palps on ventrolateral part (Fig. 2a, b), lateral antennae subulate, emerging on posterior edge of anterior lobe, similar in shape and size to palps. Posterior lobe rectangular, slightly smaller than anterior one, with median antenna, digitiform, emerging in front of caruncle, short, extending back only to first chaetiger (Figs 1b; 2a). Two pairs of eyes present. Buccal opening occupying two chaetigers (Figs 1f; 2b). Caruncle trilobed, conspicuous, about 2 times as long as prostomium, attached to and confluent with posterior prostomial lobe, free from body wall, extremity tapering, extending back to middle of fourth chaetiger, median keel broadly sinusoidal, lateral lobes plicate each with approximately 6-7 folds (Figs 1b-e; 2a), located slightly behind posterior prostomial lobe, supported by a basal plate. Pharynx unarmed, sac-like (Figs 1f; 2b).

All parapodia biramous, with thickened collars encompassing noto- and neuropodial fascicular lobes. Chaetiger 1 greatly reduced, incomplete dorsally and ventrally. Chaetiger 2 surrounding mouth posteriorly, represents first complete segmental ring (Figs 1f; 2b), with distinctly separated notopodia and neuropodia (Figs 1g, 2c); dorsal and ventral cirri conical and digitiform, respectively, both with stout basal cirrophores and slender distal cirrostyles; cirri of anterior 2 chaetigers longer than those of following chaetigers.

Branchiae present from chaetiger 4, dendritically branched, filaments densely ciliated (Figs 1h, 2a). First branchia with eight terminal filaments, best developed branchiae with 43-46 terminal filaments in 21-53 chaetigers, reducing posteriorly to four or five filaments, the last three chaetigers without branchiae.

Notochaetae coarser and shorter than neurochaetae. Notochaetae of three kinds: simple chaetae (harpoon chaetae without barbs, Fig. 2d), stout harpoon chaetae, greatly reduced in anterior chaetigers (Fig. 2e–g), well developed on following chaetigers (Fig. 2h); and slender capillary chaetae. Notoaciculae spinose, numbering 4-6 per fascicle, (Fig. 2i), arranged in row immediately in front of dorsal cirri, extending beyond chaetal lobe. Chaetiger 5 with 20 simple chaetae and few capillary chaetae; chaetiger 14 with six harpoon chaetae (with 8-9 barbs), 12 harpoon chaetae (without barbs) and 12 capillary chaetae; middle and posterior chaetigers each with 19-22 harpoon chaetae (each with about 23 coarse barbs) and 21-23 capillary chaetae. Neurochaetae of one basic kind: capillaries with or without spurs (Fig. 2j), the former with smooth long prongs 3-4 times length of short prongs (Fig. 2k–m). Neuroaciculae spinose, numbering 7-9, extending beyond neuropodial lobe, arranged along dorsal superior region of fascicle (Fig. 2n).

Paratype: Pygidium with dorsal anus opening on last three chaetigers (Fig. 1i), pygidial cirrus with rounded anal papilla.

###### Etymology.

The name of this species refers to the slender form of its capillary neurochaetae.

###### Distribution.

Presently known only from the type location, Beibu Gulf, South China Sea.

###### Remarks.

*Alleurythoe tenuichaeta* sp. n. is anatomically similar to *Eurythoe rullieri* Fauvel, 1953 because the caruncle of both species is free from the body wall. For example, the relatively narrow median keel of *Alleurythoe tenuichaeta* sp. n., does not overlap the lateral lobes, while that of *Eurythoe rullieri* broadly overlaps the contiguous lateral lobes. The new species is further differentiated in having branchiae first present from chaetiger 4, rather than chaetiger 3, and lacking the thick bifurcate neurochaetae that are characteristic of *E. rullieri* and the genus *Eurythoe* (Fauvel 1953).

*Alleurythoe tenuichaeta* sp. n. also resembles *E. paupera* (Grube 1856) in having quadrangular body form, branchiae first present from the fourth chaetiger. However, caruncles and notochaetae differ in these species. The caruncle of *Alleurythoe tenuichaeta* is attached to and confluent with the posterior prostomial lobe, and free of the body wall, while the caruncle of *E. paupera* is fixed to the first two chaetigers, extending to the anterior edge of the third chaetiger. Meanwhile, *A. tenuichaeta* sp. n. has harpoon notochaetae and bifurcate neurochaetae, both of which are absent in *E. paupera* (Grube 1856).

### Key to genera of Amphinominae Lamarck, 1818 (modified from Borda 2012)

**Table:** 

1	Caruncle absent, neuropodia arising from ventral body surface; neurochaetae retractile	***Hipponoe* Audouin & Milne Edwards, 1830**
–	Caruncle present, variably developed, neuropodia arising from lateral body surface; neurochaetae non-retractile	**2**
2	Branchiae present on all chaetigers	***Branchamphinome* Hartman, 1967**
–	Some chaetigers without branchiae	**3**
3	Branchiae present from chaetiger 6, eyes absent	***Benthoscolex* Horst, 1912**
–	Branchiae present from chaetiger 2-4, eyes commonly present	**4**
4	Chaetiger 1 dorsally continuous, complete	**5**
–	Chaetiger 1 dorsally discontinuous, incomplete	**6**
5	Stout, distally curved hooks present in notopodia of chaetiger 1; caruncle round, sessile, without free lateral wings; neurochaetae not unidentate; harpoon notochaetae with 1 row of barbs	***Paramphinome* Sars, 1869**
–	Stout, distally curved hooks not present in notopodia of chaetiger 1; caruncle stalked, broadly triangular to chordate with free lateral wings; neurochaetae unidentate; harpoon notochaetae with up to 5 rows of barbs	***Amphinome* Bruguière, 1789**
6	Caruncle small and inconspicuous, not extending beyond one external chaetiger posteriorly	**7**
–	Caruncle large and conspicuous, extending beyond one external chaetiger posteriorly	**8**
7	Branchiae present on almost all chaetigers, with modified neurochaetae	***Cryptonome* Borda, Kudenov, Bienhold & Rouse, 2012**
–	Branchiae restricted to anterior chaetigers, neurochaetae unmodified	***Linopherus* Quatrefages, 1866**
8	Caruncle with smooth median keel, with oblique divergent lateral lobes	***Pherecardia* Horst, 1886**
–	Caruncle with or without median keel, lateral lobes not oblique divergent	**9**
9	Caruncle without a median lobe, with paired lateral lobes forming a complex monopodial-like pattern of bipinnate chevrons opening anteriorly	***Hermodice* Kinberg, 1857**
–	Caruncle with a smooth median lobe, with paired lateral lobes not forming a complex monopodial-like pattern of bipinnate chevrons opening anteriorly	**10**
10	Caruncle sinusoidal, median keel not thickened, not pronounced, fused to body wall for most of its length	***Pareurythoe* Gustafson, 1930**
–	Median keel of caruncle sinusoidal, attached to and confluent with posterior prostomial lobe, supported by a basal plate and free of body wall, median keel not overlapping lateral lobes; branchiae present from chaetiger four, bifurcate neurochaetae capillary, slender	***Alleurythoe* gen. n.**
	Caruncle not sinusoidal, fused to body wall for most of its length, without basal plate, median keel thickened and pronounced, overlapping lateral lobes; branchiae present from chaetigers 1-4, bifurcate neurochaetae short and thick	***Eurythoe* Kinberg, 1857**

## Supplementary Material

XML Treatment for Alleurythoe

XML Treatment for Alleurythoe tenuichaeta
